# Energetics of Electron Pairs in Electrophilic Aromatic Substitutions

**DOI:** 10.3390/molecules26020513

**Published:** 2021-01-19

**Authors:** Julen Munárriz, Miguel Gallegos, Julia Contreras-García, Ángel Martín Pendás

**Affiliations:** 1 Departamento de Química Física y Analítica, Universidad de Oviedo, 33006 Oviedo, Spain; gallegosmiguel@uniovi.es; 2 Laboratoire de Chimie Théorique, Sorbonne Université, F. 75005 Paris, France; contrera@lct.jussieu.fr

**Keywords:** chemical bonding in real space, bond charge model, interacting quantum atoms, electron localization function

## Abstract

The interacting quantum atoms approach (IQA) as applied to the electron-pair exhaustive partition of real space induced by the electron localization function (ELF) is used to examine candidate energetic descriptors to rationalize substituent effects in simple electrophilic aromatic substitutions. It is first shown that inductive and mesomeric effects can be recognized from the decay mode of the aromatic valence bond basin populations with the distance to the substituent, and that the fluctuation of the population of adjacent bonds holds also regioselectivity information. With this, the kinetic energy of the electrons in these aromatic basins, as well as their mutual exchange-correlation energies are proposed as suitable energetic indices containing relevant information about substituent effects. We suggest that these descriptors could be used to build future reactive force fields.

## 1. Introduction

Computational and theoretical chemistry (CTC) has undoubtedly come of age. Advances in both electronic structure methods and computer technology now allow researchers to perform simulations of realistic systems with outstanding accuracy within affordable times, so that CTC is already playing a crucial role in fields like big pharma industry or materials design [1,2,3,4,5,6]. Machine learning (ML), on the other hand, has fully revolutionized the field [7,8], and problems which were once thought to be out of reach in a foreseeable future, like protein folding, are close to being solved [9]. Given this favorable scenario, it should never be forgotten that the simulation of complex systems, or the training of deep neural networks, rely at one point or another on human understanding of an underlying energy model, be it a well parameterized force field (FF) in the first case, or a specific neural network (NN) in the second. Many successful FFs or NNs proposed in the CTC context share a common structure rooted in some of the most cherished chemical concepts: atoms that form molecules through well-defined spring-like chemical bonds, which can be formed or broken [10]. Although the formation and rupture of these entities that chemists call ‘bonds’ lies at the core of chemical intuition, the formal construction of atomistic models has usually followed, perhaps as a result of historical precedence, the physicist’s viewpoint, based on a many body expansion of the energy landscape. Early atomistic models [11] already introduced the idea that pairwise additive pair potentials were essentially enough for simulation purposes, with smaller three body corrections that could be added ad hoc, if necessary. This mantra was inherited in the first wide purpose force fields and has remained practically untouched to date.

The mismatch between the bond-centric chemical and the atomistic points of view has led to several aberrations, which may well be exemplified by the concept of atom types. When atoms are used as the FF building blocks, specific parameterizations for different coordination or bonding environments are needed. Up to ten different types of nitrogen atoms exist in the generalized Amber FF (GAFF) [12], for instance. The situation in ML-based methods is not better, since NNs normally inherit the atom types logic, although the actual number of types is not fixed, but inherited from the information provided for the training of the network. It is clear that much of this zoo could be avoided if electron pairs, not atoms, were selected as the energy model building blocks, as it is electrons (i.e., bonds) that rearrange in the course of a reaction. Moreover, the direct use of electron-pair units (which behave like bosons) may decrease the complexity of the parameterization, much influenced by the Fermi statistics of electrons.

We very much think that such an approach is worth pursuing, and we have proposed in this regard to use real space partitioning techniques that are able to isolate electron-pair regions and to couple them to real space energy partitioning methods [13,14]. This way, we aim to find how well these techniques are able to recover known chemical insight and to move slowly toward bond-centric energy landscapes that can offer accurate results at a much smaller and less arbitrary parameterization effort.

In a previous work [14] we have shown how the abovementioned coupling recovers and justifies the bond charge model (BCM) proposed by Parr and Borkman [15,16]. To do that we exploited the topology induced by the Electron Localization Function (ELF) proposed by Becke [17], given its ability to define electron pair regions in real space [18], and its good performance in the understanding of chemical properties and structure [19,20], as well as in reactivity [21,22]. We coupled the ELF with the interacting quantum atoms (IQA) approach [23,24], a powerful real-space quantum-chemical topology tool that allows partitioning the total energy of a system into chemically meaningful contributions, and has also been successfully applied in the analysis of a number of chemical phenomena from an intuitive chemical bond perspective [25,26,27]. With this methodology, it was shown that all kinetic, electrostatic, and exchange-correlation terms for the electron pairs follow straightforward scaling laws that were proven to be valid in widely different bonding regimes, ranging from homopolar to highly polar. It was clearly shown that the original BCM model, as well as its updated version, in which it is coupled with the ELF [28], works well in as much the quantum mechanical correction represented by the exchange-correlation term is considerably smaller than all the other contributions entering into the BCM energy. In this sense, it is remarkable to note that this model has recently been applied to the description of a variety of C–C bonds [29].

Kinetic, electrostatic and exchange-correlation terms have been shown to follow distance dependencies that are akin to straightforward scaling laws. Electron pairs, as described by the ELF, can be modelled from the homogeneous electron gas point of view. The proposed scaling behaviors have proven to work reasonably well for different polarities (from homonuclear to highly polar bonds, including non-conventional bonds). Whenever the electron pair fails to describe bonding, like in metallic bonds, or when highly delocalized bonding regimes set in, the model is expected to fail. We have also shown how simple local measures can be introduced to identify these situations [14].

Application of the IQA-ELF methodology (the combination of IQA energy decomposition scheme with the ELF topology in the BCM framework) is still in its infancy, although the ease with which it deals with otherwise difficult-to-model entities, like lone pairs, encourages us to progress in its development. As a further step in such an enterprise, we have decided to invest our efforts in checking to what extent it may be able to recover more subtle electronic effects, particularly those which are usually explained from an atomic-based rather than from a bond-based point of view. Out of the many possible examples out there, we have decided to study the effect of substituents in electrophilic aromatic substitutions (EAS) [30]. These are thoroughly studied reactions, characterized by strong orientation/activation effects triggered by the nature of the substituents [31,32]. As every fresh organic chemistry student learns, both inductive as well as resonance or mesomeric components are usually invoked to rationalize the experimentally observed behavior. Rules to predict the *ortho*-/*para*- or *meta*-orienting ability of substituents are an active topic in chemical research and have been recovered from almost every imaginable point of view [33,34,35,36,37,38,39,40,41]; and even the EAS reaction mechanism is still under debate [30,42,43,44,45,46,47]. Among the various previous approaches for EAS study, we are especially interested in the ELF due to its direct relation with the BCM model and the IQA-ELF coupling. Notice that the ELF application to this kind of processes was first addressed by Silvi and coworkers [48,49,50], who were able to predict the most reactive position in terms of an ELF-based reactivity index in mono-substituted benzene molecules, and then extended it to polyaromatic molecules and to aromatic heterocycles.

Since standard wisdom is atom-centric, here, we want to examine whether the bond-centric IQA-ELF point of view is able to provide further insights into this problem. In this first incursion we will just examine IQA-ELF quantities in isolated substituted benzene rings, much in the light of previous treatments performed within the conceptual density functional theory framework [48,51].

An interesting point regards the absence of σ-π channels in the orbital invariant treatments provided by real space techniques, and, for this distinction, it becomes essential to isolate inductive (I) from mesomeric (M) effects [52]. In this sense, it is relevant to recognize that, in the last decade, a number of works have clearly shown that we can relate these chemical concepts to the spatial decay rate of several electronic reduced density matrices. The exponentially decaying nature of delocalization measures in insulators becomes linked to Kohn’s shortsightedness of the one-particle density and to short-range inductive effects, while the generally oscillating and geometrical decay rate of this quantities in metallic-like systems is connected to long-range mesomerism [53,54]. It will be shown that we can recover the classical insights about I and M effects from the decay of IQA-ELF quantities with the distance to the substituent.

## 2. Results and Discussion

**Figure 1:**
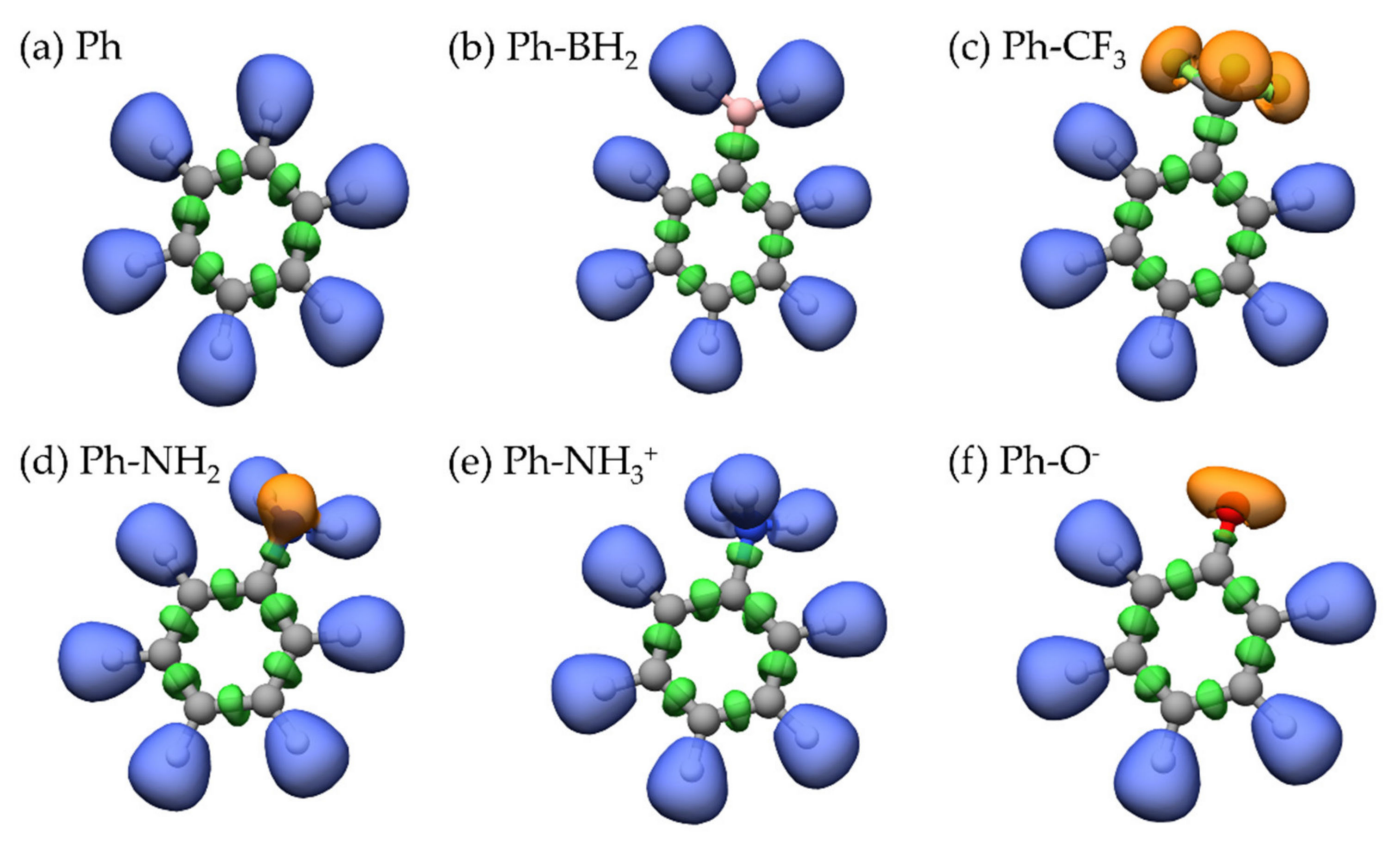
ELF localization domains (*η* = 0.8) for six (**a**–**f**) Ph–X compounds that cover several I/M electronic effects scenarios. Notice that the aromatic ring is structurally stable with respect to changes in the X substituent. In this work, we focus on the V(C_i_,C_i±1_) disynaptic valence domains, shown in green.

Table 1 contains a succinct list of X substituents classified according to their activating/deactivating nature as well as to their orienting ability [31]. Its purpose is illustrative, and we will refer to it to contextualize some of our findings. We start by considering briefly the topology of the ELF gradient field, which is shown in Figure 1 for some representative examples. Notice that the results are shown in a graphical manner for the sake of clarity, while the numerical data and some alternative figures are provided in the Supplementary Materials.

### 2.1. ELF Topology

As originally put forward by Fuster et al. [48], monosubstitution of benzene does not alter the basic ELF topology of the aromatic ring. The phenyl subspace displays the same number and type of basins as benzene, with standard core regions for all carbon atoms, C(C_i_) i = 1,6, as well as V(C_i_,H) and V(C_i_,C_i±1_) disynaptic valence domains (note that the list i = 1.6 is circular, so C(C_1_) is linked to V(C_1_,C_2_) and V(C_1_,C_6_)). In this sense, the ELF field is structurally stable against electrophilic aromatic substitution, easing the comparison of Ph–H (Figure 1a) with Ph–X derivatives (see Figure 1b–f for 5 substituted systems). Substitution does alter the phenyl subspace bifurcation tree, and two kinds of diagrams were found according to the nature of the X substituent that could resolve *ortho*/*para* from *meta* orienting moieties [48]. Although this analysis showed how the perturbative approach that examines the electronic structure of the reactant alone can be correlated to the observed experimental behavior, it did not offer any clear physical rationalization of it. In order to offer such a link, it is advisable to turn to basin observables, be they based on electron populations and their fluctuations or, as we will show, on IQA-ELF quantities.

### 2.2. Recognizing Induction and Mesomerism

**Scheme 1:**
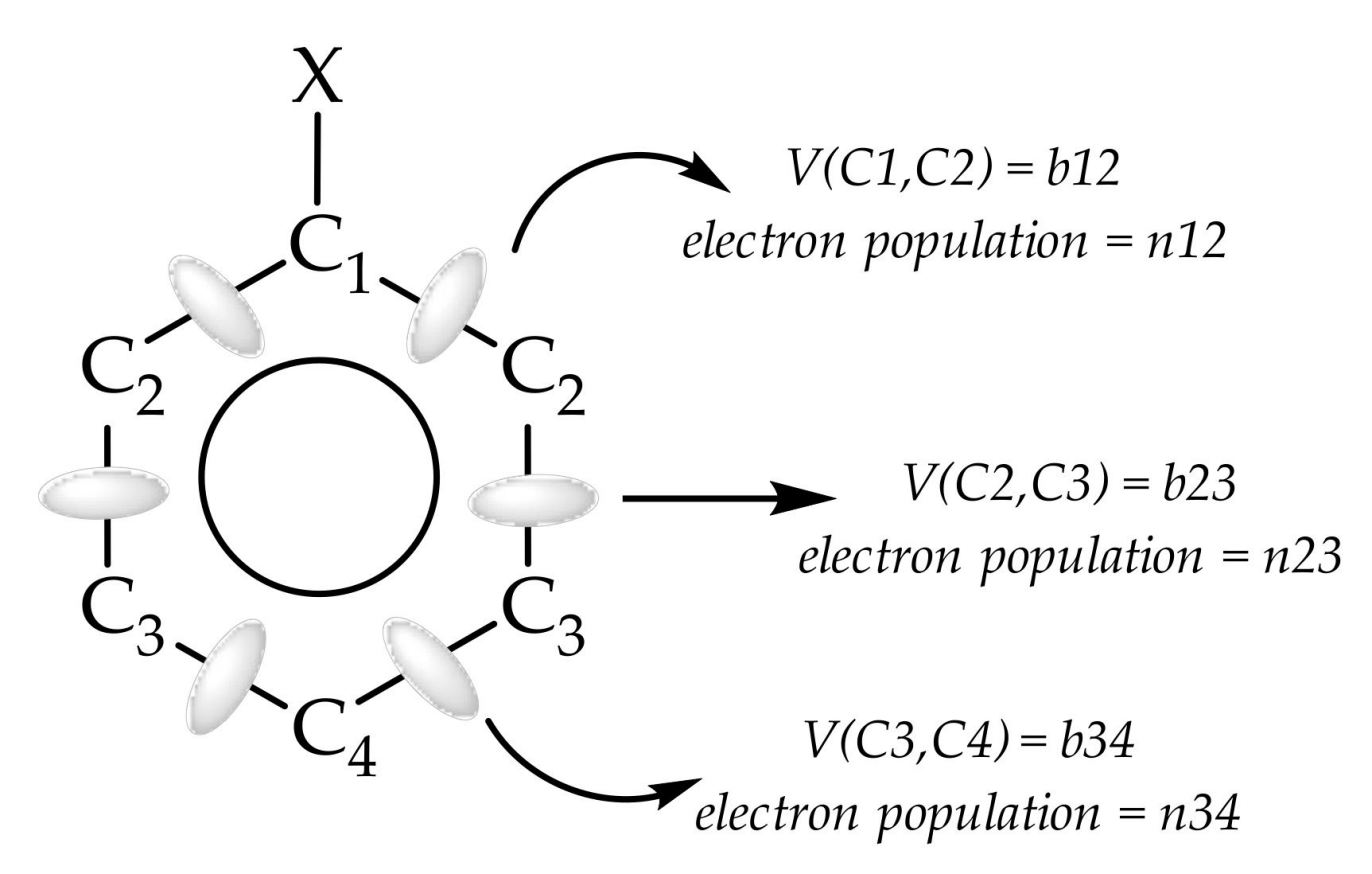
Schematic representation of the nomenclature for C atoms, *bij* disynaptic ELF basins and their electron populations (*nij*) considered in this work. Notice that we have considered a symmetric system.

**Figure 2:**
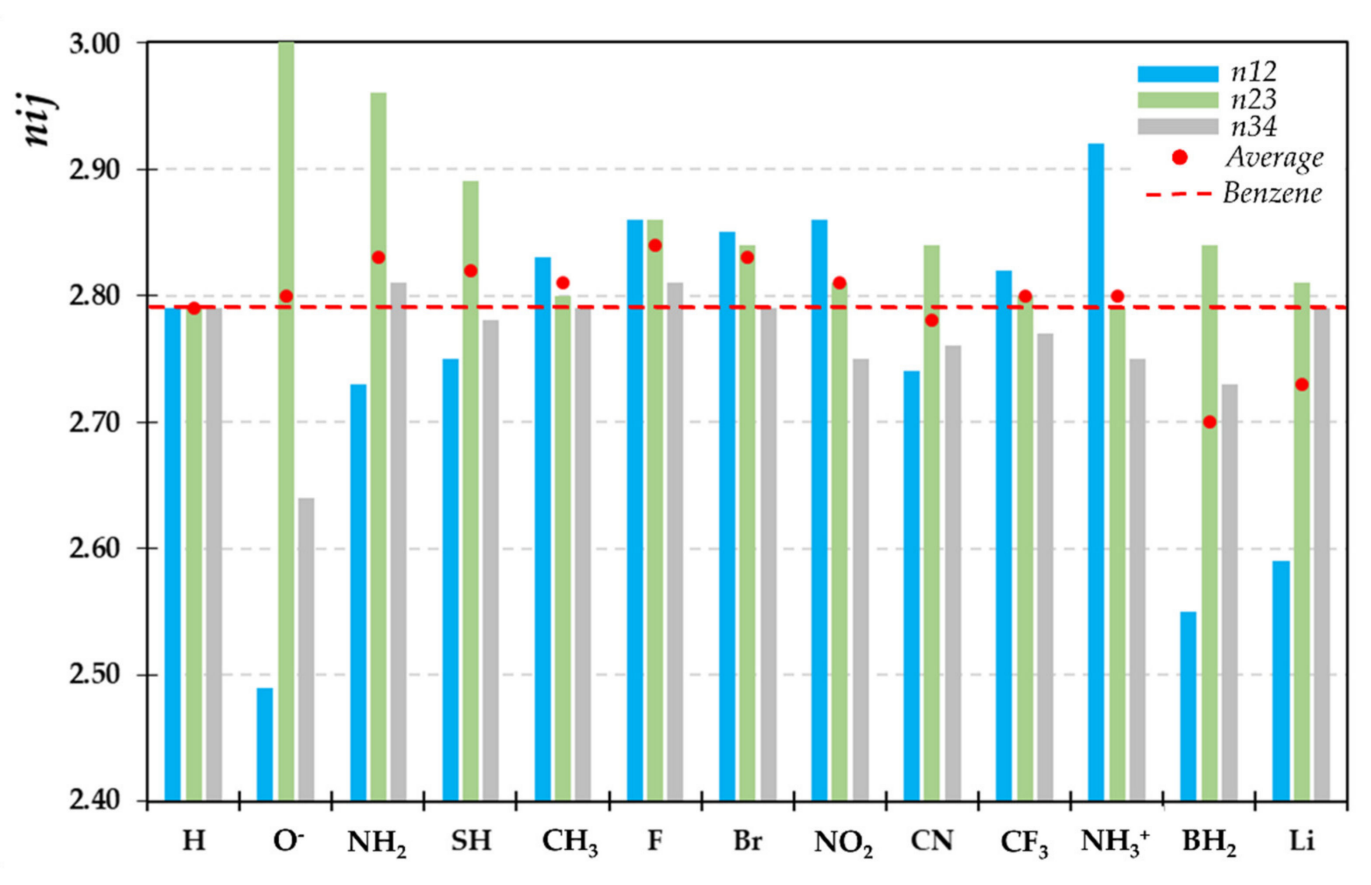
*bij* basin populations (*nij*) for the selected set of Ph–X systems. All data in au. Notice that *n23* for X = O^−^ is equal to 3.29, but it has been cut at 3.00 for visual purposes.

It is interesting to check how inductive and mesomeric effects can be detected in a bond-centric decomposition such as that provided by the ELF field. A number of basin expectation values can be used in this regard, although the simplest one is, no doubt, the basin electron population. Since we are mainly interested in changes in the aromatic ring, we will only consider V(C_i_,C_i±1_) populations in the following. Let C_1_ be the substituted carbon atom, and let us label the V(C_i_,C_i+1_) = V(C_i_,C_j_) bond basin and its electron population as *bij* and *nij*, respectively, as depicted in Scheme 1. Figure 2 gathers some representative basin populations across the ring, while a full list can be found in Supplementary Table S1. We will base our arguments in the following paragraphs on the results found in this figure, although they are general to all the systems we have studied. Notice that in less symmetrical substituents (like Ph–SH), nearly equivalent basin populations like *n23* and *n56* were found in some cases to be slightly dissimilar and thus averaged. The same procedure was applied to kinetic and exchange-correlation energies and delocalization indices.

Most of the chemical knowledge regarding the electron density accumulations or depletions that the X substituent induces in the aromatic ring should be found in the *nij* populations. Both the carbon cores and the V(C,H) basins do surely show variations with X, but these are not directly related to ring electron delocalization. Our data are mainly in line with standard wisdom, although several interesting anomalies stand out.

In the first place, it is rather clear that, a bit unexpectedly, the total number of electrons in the aromatic ring, which can be measured by the average *nij* population (red spot in Figure 2), is generally larger than that found in the unsubstituted benzene (red dashed line), see Supplementary Figure S1 for *nij* relative to benzene (Δ*nij*). It is only smaller with strong EWGs, like BH_2_ or CN, but not in the case of strong deactivating groups like NO_2_ or in weaker ones like the halides. There is indeed a tendency toward larger/smaller total populations in the case of activating/deactivating groups, but a loose one. This warns about the blind use of arrow pushing techniques, which dominate textbook explanations.

A salient feature in Figure 2 is the very different behavior of the population of the bond basins with X. On the one hand, we find systems like toluene, Ph–CF_3_, or Ph–NH_3_^+^, for which the substituent effect decays with the distance, *n12* > *n23* > *n34*. This extinction of the density perturbation induced by X as the distance to the substituent increases agrees with an insulator-like behavior of the one-particle density matrix, which we here associate to (dominant) inductive effects. Notice, however, that the methyl group, usually classified as a weak EDG, leads to a distribution not so far from that found for CF_3_, which is however a traditionally strong EWG. It is also clear that large inductive effects lead to stronger perturbations, and that *n34* falls well below the reference Ph–H value in Ph–NH_3_^+^, for instance. Activating groups tend to display smaller *n12* values (in the case of O^−^, *n12* = 2.49 *e*), while deactivating groups usually show larger *n12* populations, peaking at 2.92 *e* for Ph–NH_3_^+^.

**Scheme 2:**
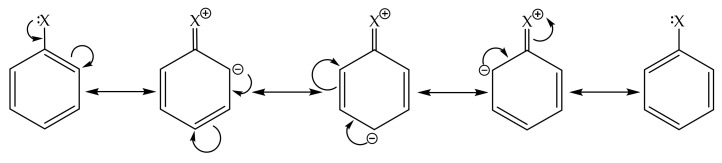
Schematic representation of the resonance structures of a +M substituent group X donating a lone pair to the aromatic ring. A similar pattern is obtained when X has a π-hole, now with positive formal charges in the ring.

A very different kind of distance behavior is also obvious. In many of our systems, the *nij* values oscillate, and *n23* is maximum. A comparison of Table 1 and Figure 2 (or Supplementary Table S1 for the full set of molecules) leads to identifying these systems as those where mesomeric effects are important. Interestingly, molecules displaying +M and −M classical resonance give rise to the same kind of oscillatory evolution, with large *n23* and smaller *n12* and *n34* populations. In the +M case, *n23* tends to be clearly larger than in the −M one, peaking at *n23* = 3.29 *e* when X = O^−^. For both +/−M systems a clear tendency to a *n12 < n34* configuration is also evident. This behavior is relatively consistent with the naïve resonance structures that include a C_1_ = X double bond, thus reinforcing the quinoid-like C_2_–C_3_ links in most resonance forms, at the expense of the C_1_–C_2_ and C_3_–C_4_ ones (see Scheme 2).

Halogens display a peculiar behavior. On the one hand, their ring bond populations decay with the distance, pointing to dominating inductive effects. On the other, their average number of electrons is quite large, in line with other activating systems in the upper part of Table 1. Fluorine, in particular, is close to showing a mesomeric-like maximum at *n23* that points toward its standard classification as a π-donor.

The nitro group also deserves some specific comments. Despite being usually classified as a strong −I/−M deactivating group, in the same category as CN, our data does not find any (dominant) mesomeric effect, but a behavior much more in line with other −I deactivating groups such as CF_3_ or NH_3_^+^. Cyanide, on the contrary, does clearly show an oscillating M pattern. Given that the −M classification of NO_2_ is based on the purported positive π hole of the N atom in the NO_2_ Lewis structure, something which is not supported by real space chemical bonding analyses, we think that the present results might be better representing the true nature of the nitro group deactivating features. When the π-Lewis acid nature of a group is out of question, as in the BH_2_ case, the oscillations are clearly present.

Finally, Ph–Li is also interesting on its own. The Li atom has a very low electronegativity and no π density channels, basically leaving a Ph^−^ anion characterized by a very small aromatic population that responds mesomerically to the perturbation. Actually, its average number of aromatic bond population is the second lowest after BH_2_.

As already commented, these observations are compatible with knowledge about how density perturbations decay with the distance in molecular systems. As proven by Walter Kohn in a seminal paper [55], insulators are characterized by an exponential drop of electron-electron correlations with the distance, which ends up in the non-diagonal elements of the one-particle density matrix (1DM) also decaying exponentially, $\rho \left(r;r+s\right)\approx e^{-\eta s}$. On the contrary, the 1DM falls algebraically in metals, $\rho \left(r;r+s\right)\approx as^{-d}$, where the exponent *d* depends on the dimensionality of the system. This difference supports the textbook chemical picture of localized/insulating, delocalized/metallic electrons. We have also shown [53,54] that the algebraic decay is found in unsaturated alternant hydrocarbons, where an overall inverse quadratic envelope is enriched by an oscillatory pattern of the real space delocalization indices (DI), which measure the fluctuation of the electron populations in two different spatial regions, or the number of shared electrons between them. It was found that when atomic regions are used, the DI(A,B) values with A and B separated by an even number of bonds (grossly corresponding to meta pairs) vanish in the tight-binding approximation, and that they turn out to be much smaller than DIs between pairs separated by an odd number of bonds in actual calculations.

### 2.3. Bond Delocalization and Mesomerism

**Scheme 3:**
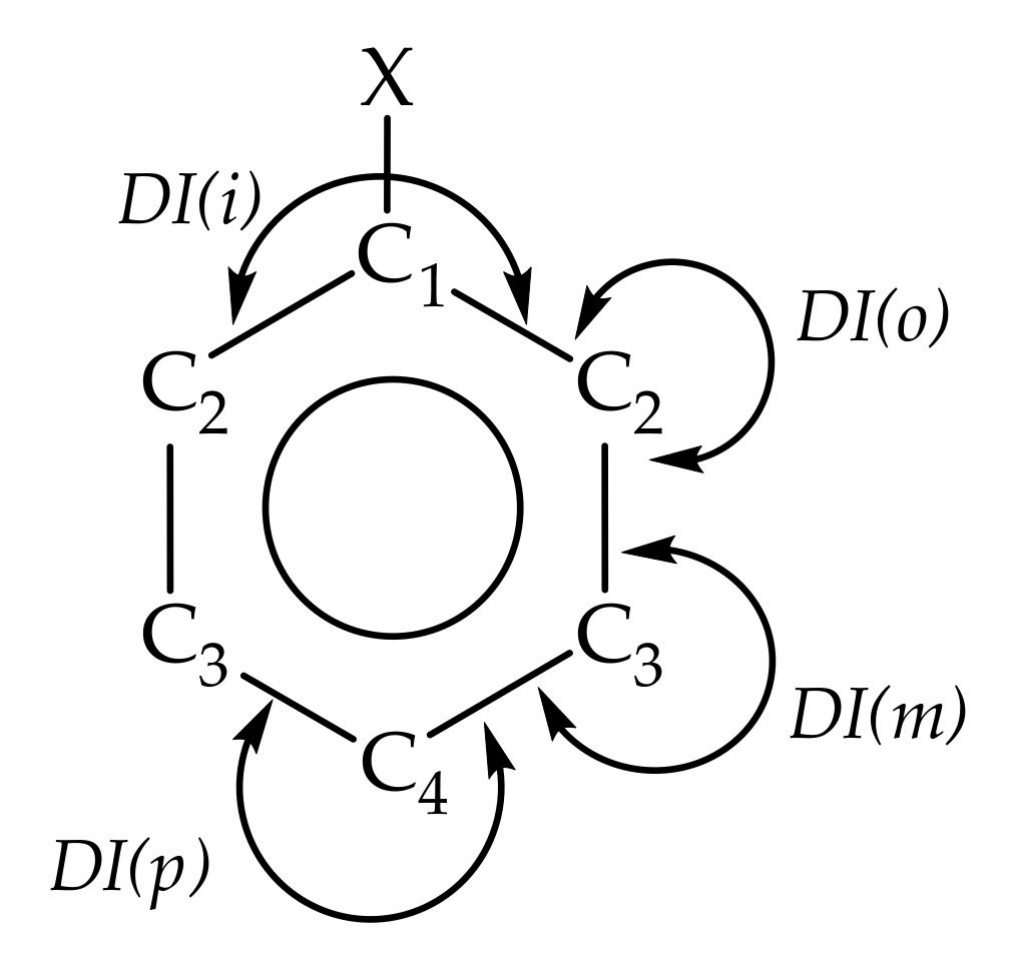
Schematic representation of the nomenclature for DIs(*a*) considered in this work. Note that we have considered a symmetric Ph–X system.

**Figure 3:**
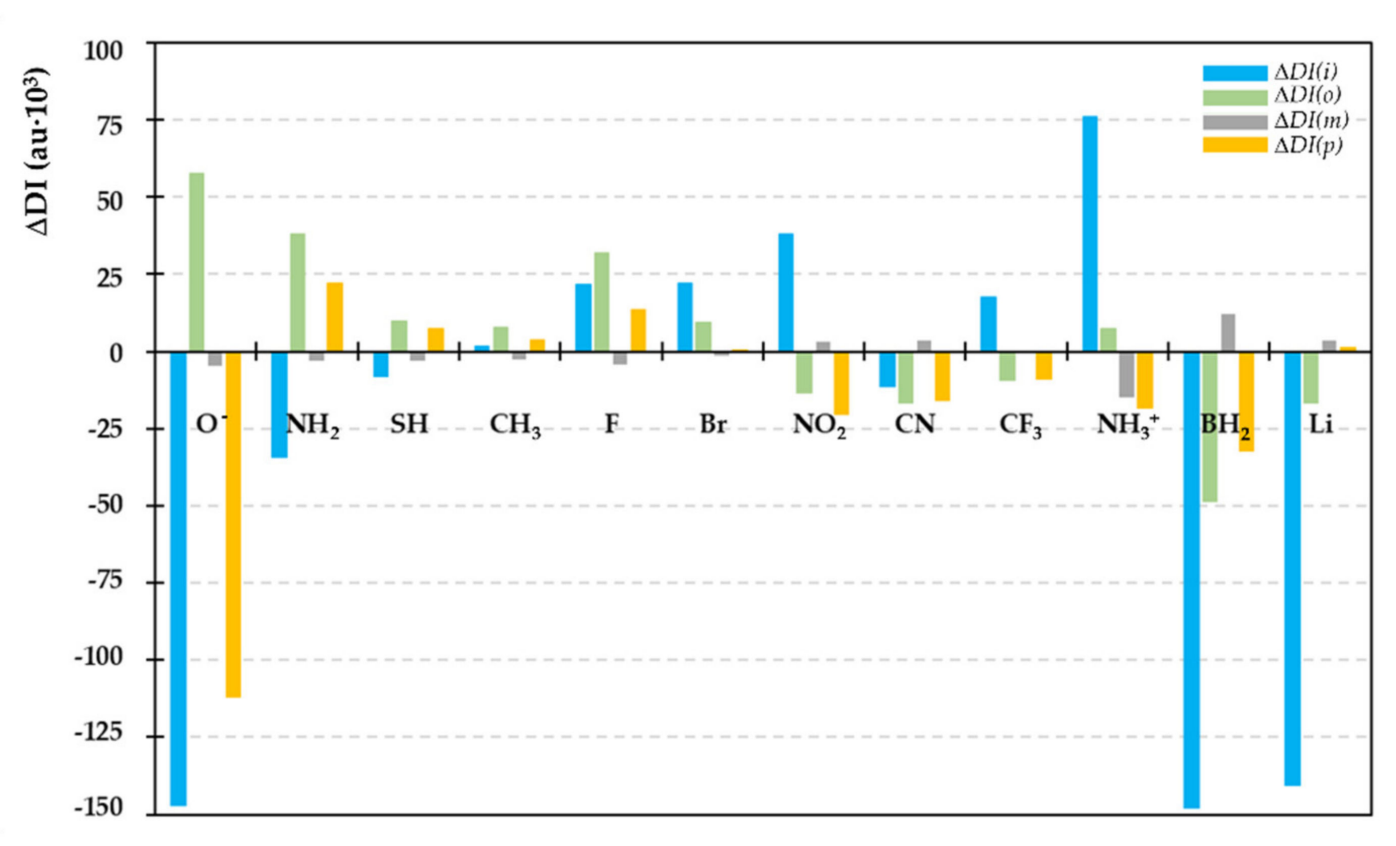
ΔDI(*a*) for the set of selected systems. See the text for a description. All data in au × 10^−3^, referred to the vicinal DI in benzene, equal to 0.555 au: ΔDI(*a*) = DI(*a*,X) − DI(*a*,X=H).

The findings in the previous subsection have prompted us to examine the ability of the different aromatic valence basins to delocalize their electrons among themselves as a way to rationalize the substituent effects in electrophilic attacks. To do so, we examine vicinal DIs, i.e., delocalization indices between adjacent V(C_i_,C_i+1_) and V(C_i+1_,C_i+2_). These DIs can be identified by the common C_i+1_ atom, which may correspond to either the substituted position, ipso, or to the ortho, meta, or para ones, as shown in Scheme 3. We will thus refer to them as the DI(*a*) descriptors, with *a* = *i*,*o*,*m*,*p*. A summary of our data for the same set of selected systems as for Figure 2 is gathered in Figure 3. Values are collected relative to the unsubstituted benzene molecule in order to ease the analysis in terms of substitution induced enhancements. The full set of results can be found in Supplementary Table S2.

Before performing a detailed analysis of the data, it is relevant to point out that the DI(*a*) descriptors do have a clear physical meaning. They exactly measure how correlated the populations of vicinal aromatic bonds are, since DI(A,B) = −2·cov(*n_A_,n_B_*), with A,B being two spatial domains, *n_A_,n_B_* their respective electron populations, and cov(*n_A_,n_B_*) the covariance of these populations. In other words, the DI(*a*) descriptor tells us about how easily electrons traverse the C_A_ atom, flowing through it. Since in the electrophilic attack of the C_A_ moiety the arenium ion strongly modifies its vicinal links, we expect ΔDI(*a*) = DI(*a*,X) − DI(*a*,X = H) to correlate with some of the experimental results.

A quick look at Figure 3 shows that this is indeed the case. First, we mention that these descriptors are not completely independent from those in Figure 2. For instance, ΔDI(*i*) is proportional to *n12*, and whenever the latter population is larger than the one in benzene, ΔDI(*i*) is positive and vice versa. This is to be expected, since the number of shared pairs of electrons depends on the basin populations. Again, the importance of mesomeric effects for a substituent can be grasped from the data through a negative ΔDI(*i*). When inductive effects dominate, ΔDI(*i*) is positive, and this again includes the nitro group.

Let us now focus on the changes in ΔDI(*i*,*o*,*m*,*p*). First, classical activating groups such as NH_2_, SH, or CH_3_ display a +,−,+ pattern for the *o*,*m*,*p* descriptors, respectively (see Table 1). This corresponds to a greater delocalizing ability than plain benzene at the *ortho* and *para* sites, while a smaller one at the meta position. Notice that the first two groups are classified as −I/+M, while the methyl substituent is usually understood as a pure +I substituent. Although the magnitude of ΔDI(*i*) is related to the strength of the substituent effect, care has to be taken due to the influence of the underlying basin populations. For instance, fluorine is found to be *ortho*/*para* directing quite as much as NH_2,_ but its larger basin populations (Figure 2) mask its strength. A suitably normalized descriptor might solve this issue, but has not been pursued in this contribution. We do not skip the fact that the oxido group displays a large negative ΔDI(*p*) value. We do not have a fully consistent explanation for this fact, but as in the case of the nitro group behavior, we tend to think that the traditional classification may hide other interesting effects that should be further investigated.

Similarly, deactivating groups display negative ΔDI(*o*,*p*) values, with *meta* contributions either positive or negative. The change from a +,−,+ to a −,+,− pattern as we change X is very interesting, and demonstrates that bond-centric descriptors do also pick up a coherent image of the electronic effects under study. X = CF_3_ (and also CHF_2_, or CH_2_Cl, see Supplementary Table S2) displays triply negative values, BH_2_ a considerable enhancement of the meta index, and the NH_3_^+^ group a small activation of the ortho position, which might be masked in a true electrophilic attack due to electrostatic repulsion with the attacking moiety.

**Figure 4:**
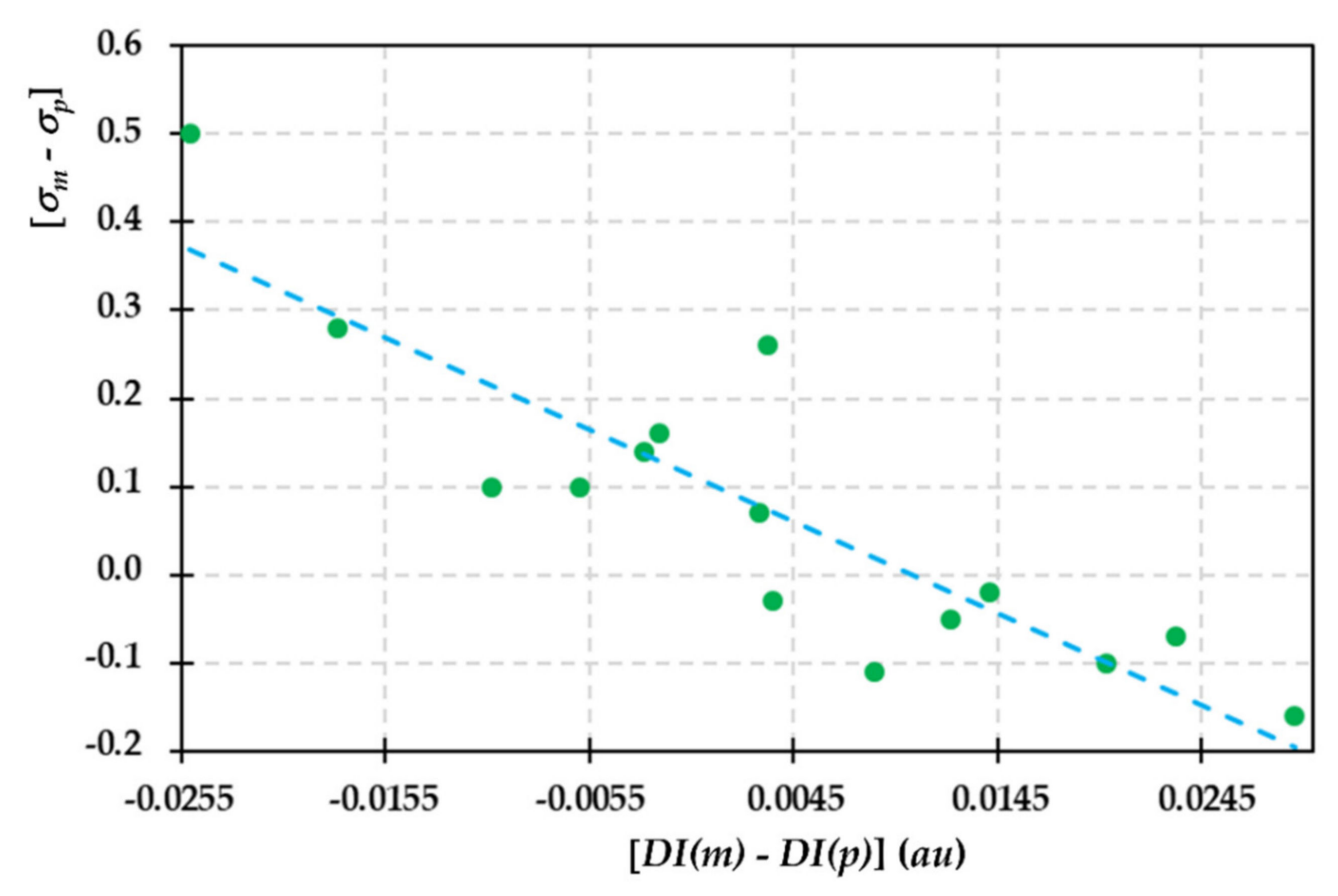
Correlation between *σ_m_ − σ_p_*, as taken from Ref. [56], and DI(*m*) − DI(*p*), as calculated in this work. The linear regression coefficient is R^2^ = 0.7649. The oxido group has been excluded from the data as it deviates quite a lot from the general tendency.

The experimental substituent effects have been usually rationalized by means of the Hammett equation, in which ratios of reaction rates are interpreted in terms of *σ* and *ρ* constants which consider substituent and reaction conditions effects, respectively. Figure 4 shows how σ*_m_* − σ*_p_* correlates reasonably with DI(*m*) − DI(*p*) for a number of systems. The Hammett constants have been taken from Ref. [56]. Similar correlations were found through local ELF bifurcation values [48], demonstrating that our physically based descriptors are able to capture, at least grossly, the subtleties of standard organic chemistry knowledge.

### 2.4. IQA-ELF Descriptors

We have shown how basin electron populations and fluctuations can be used to offer a consistent picture of inductive and resonance effects without an explicit σ − π separation. We now turn to the less studied basin energetic expectation values. We will start with the kinetic energy. As in previous works, we use the positive definite kinetic energy density, see Equation (1).

(1) $$t=\frac{1}{2}\nabla \cdot \nabla^{\prime }\rho \left(r;r^{\prime }\right)$$

For only in the case of QTAIM basins we can use any Laplacian based kinetic energy density. This is a common practice, and avoids possible negative basin expectation values.

**Figure 5:**
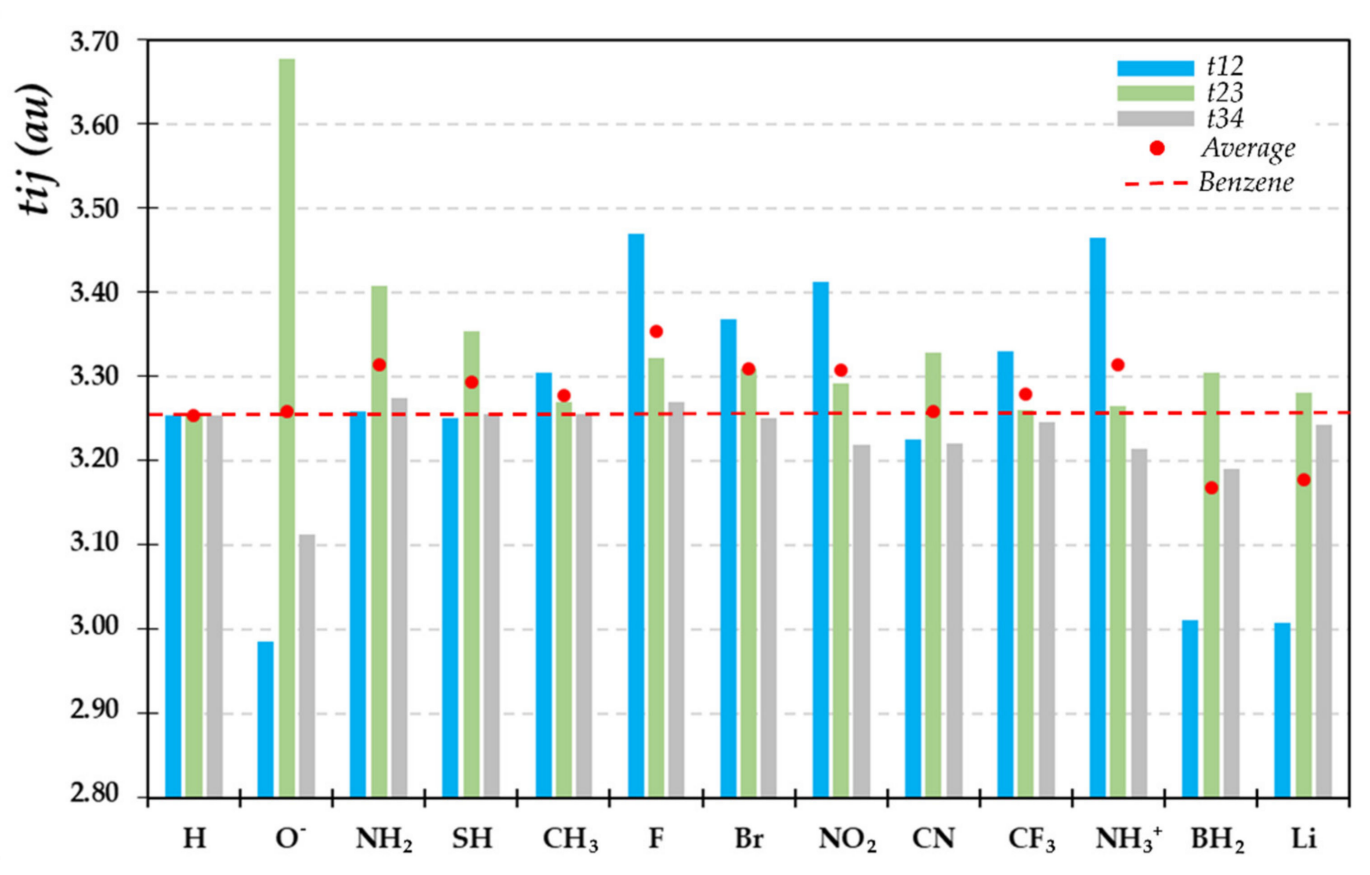
*bij* basin kinetic energies (*tij*) for a set of selected Ph–X systems. Notation as in Scheme 1. All data in au.

Kinetic energies of the aromatic valence basins are collected in Figure 5 (and Supplementary Table S3 for the complete set of systems), with a notation equivalent to that of Figure 2 (provided in Scheme 1), and the kinetic energy with relative to benzene (Δ*tij*) is shown in Supplementary Figure S2. As expected, the basin kinetic energies are proportional to the basin populations, so that as a first approximation, Figure 2 and Figure 5 show similar trends.

More interesting is the consideration of the domain kinetic energy per electron, which can be constructed immediately from the data. This is 1.166 au in benzene, and deviations from this result are quite small in all cases. A detailed analysis shows that *t23*/*n23* and *t34*/*n34* are almost unaffected by substitution, and that the largest variations are found in *t12*/*n12* which is larger than in benzene. This indicates that the perturbation of the nodal structure of the wavefunction, or of the curvature of the 1DM, falls quite fast with the distance to the substituent. For instance, $\Delta \left(\frac{t_{12}}{n_{12}}\right)$ is equal to 0.033, 0.028, and 0.027 au for the oxido, amino, and nitro X moieties, respectively.

**Figure 6:**
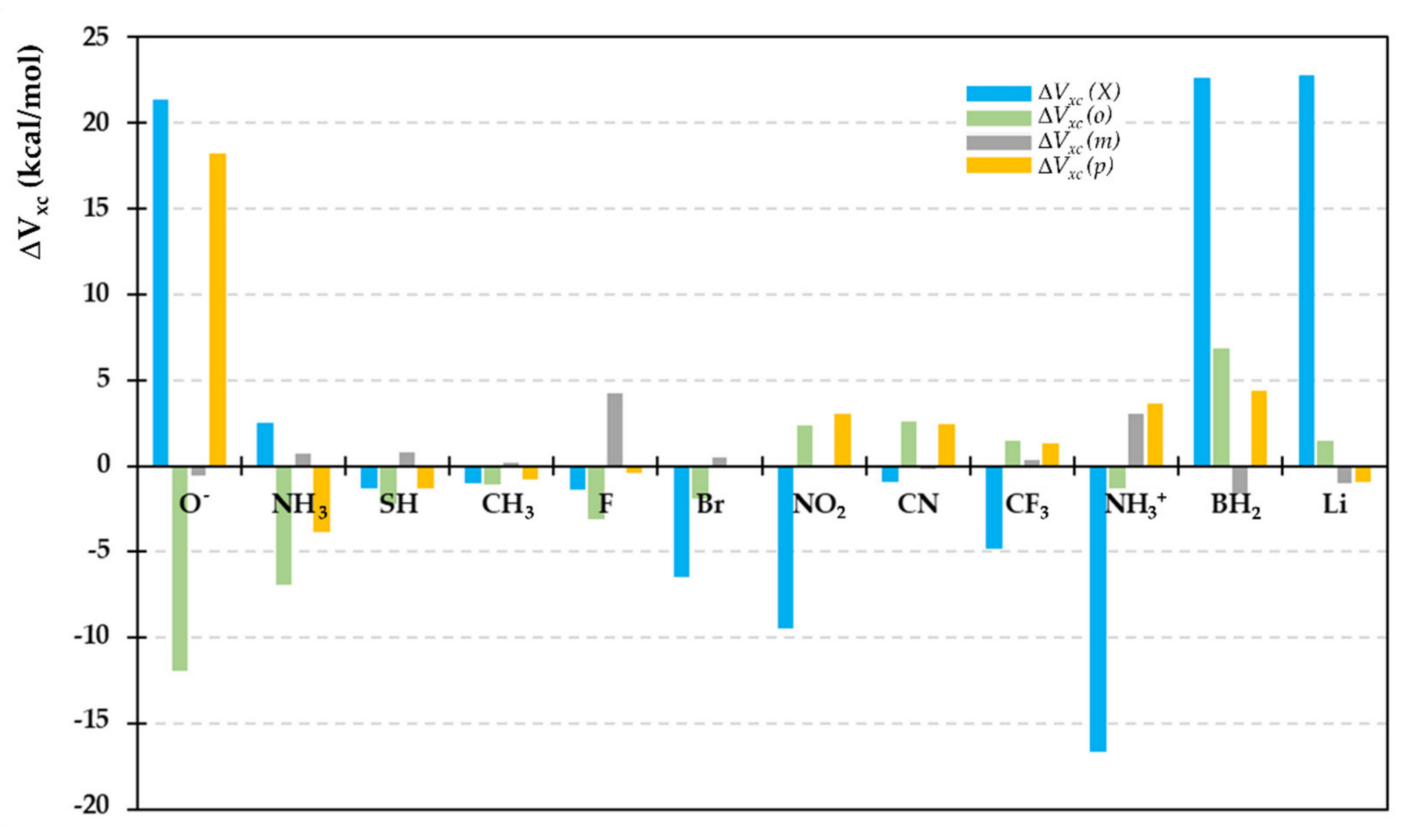
Vicinal exchange-correlation energies relative to benzene (Δ*V_xc_*) in the set of selected systems. See the text for a description. All data in kcal/mol, referred to the vicinal *V_xc_* value in benzene, equal to −100.4 kcal/mol.

Finally, the only remaining IQA-ELF energetic parameter that we expect to be related to orientation effects in electrophilic aromatic substitutions is the inter-basin exchange-correlation energy, $V_{xc}^{AB}$. Electron-nuclear attractions are density dependent quantities that are not expected to respond clearly to X effects, so they will be skipped in this first contribution. As repeatedly shown [25], *V_xc_* is a measure of the covalent energy associated to the interaction between the electrons contained in domains A and B. For the sake of clarity and given its direct connection with the delocalization index, we represent *V_xc_* for the pairs of basins under study with respect to benzene (Δ*V_xc_*) basins in Figure 6 (see Supplementary Table S4 for the numeric values of the complete set of molecules). We again label the interaction by the common carbon atom in the adjacent bond basins, as indicated in Scheme 3 for delocalization indices.

As it can be immediately checked, exchange-correlation energies mimic population fluctuations [57] and thus inter-basin electron delocalization. It is particularly interesting that the energy descriptors we have investigated in this contribution follow rather closely their population analogs. On the one hand, kinetic energies are found to evolve as populations. On the other, exchange-correlation energies can be sensed by electron delocalization indices. This is of considerable importance since the computation of energetic basin observables, particularly in the case of two-electron ones, is extremely computationally intensive. In the pseudo one-determinant approximation that we are using here, for instance, the calculation of *V_xc_* requires 6D numerical grids, that can be computed with O(*N*^4^) effort if bipolar expansions are used [58]. Meanwhile, the calculation of delocalization indices is a simple byproduct of the construction of the domain overlap matrices for the set of occupied orbitals, just needing 3D grids.

Overall, we think that building substituent-responsive force fields based on electron-pair energetic descriptors is within reach, for the IQA energetic domain expectation values have been shown to capture the essentials of the well-known, nevertheless subtle inductive and mesomeric effects in the simplest electrophilic aromatic substitutions.

## 3. Theoretical and Computational Details

All electronic structure calculations and wavefunctions for ELF and IQA analyses were obtained through DFT calculations, via the Gaussian 09 suite (Gaussian, Inc.; Wallingford, CT, USA) [59]. The B3LYP exchange-correlation functional [60,61], in conjunction with the 6-31G(d) basis set [62,63] were used. The method selection was validated in Ref. [14], with respect to the basis set and calculation method.

In order to obtain the domains associated to bonding and non-bonding pairs, we used the Electron Localization Function (ELF) [17] in its simplest formulation. The ELF kernel, $\chi_{\sigma }$, which was originally defined in terms of the curvature of the Fermi hole [64], can be interpreted as a measure of the excess of local kinetic energy due to the Pauli Principle, relative to a homogeneous electron gas kinetic energy density [65]. The ELF, *η*(*r*), is then obtained by mapping its kernel, $\chi_{\sigma }$, through a Lorentzian (see Equation (2)) in order to obtain a function that ranges from 0 to 1.

(2) $$\eta \left(\vec{r}\right)=\frac{1}{1+\chi_{\sigma }^{2}}$$

This scalar field is used to induce an exhaustive topological partition of space into non-overlapping domains (basins) whose properties can be determined by integrating appropriate operator densities over their associated volumes. When the electron density, *ρ*(*r*), is integrated, for instance, the average number of electrons in the domain is recovered. ELF basins have been calculated through the TopMod program [66], with a tridimensional grid of 150 points in each direction. The energetic descriptors of these topological basins have been calculated within the IQA energy decomposition scheme [23]. IQA is usually applied to the QTAIM atomic decompositions, leading to Equation (3).

(3) $$E=\underset{\sum_{A}E_{intra}^{A}}{\underset{︸}{\underset{A}{\sum }\left(T_{A}+V_{ee}^{AA}+V_{en}^{AA}\right)}}+\underset{\sum_{A>B}E_{inter}^{AB}}{\underset{︸}{\underset{A>B}{\sum }\left(V_{en}^{AB}+V_{ne}^{AB}+V_{nn}^{AB}+V_{ee}^{AB}\right)}}$$

In this expression we sum over atomic regions, and the total energy is decomposed into intra-atomic contributions, $E_{intra}^{A}$, and inter-atomic interactions between each pair of atoms, $E_{inter}^{AB}$. The former is the sum of the kinetic energy of electrons in atom A, $T_{A}$, and the electron-electron and electron-nucleus interactions, $V_{ee}^{AA}$ and $V_{en}^{AA}$, respectively. $E_{inter}^{AB}$ is obtained by adding the interaction among the electrons in A and the nucleus of B, $V_{en}^{AB}$; its BA symmetric counterpart, $V_{ne}^{AB}$; and the nucleus-nucleus and electron-electron interactions between A and B, $V_{nn}^{AB}$ and $V_{ee}^{AB}$, respectively. The electron repulsion terms are usually divided in IQA into a classical contribution that depends only on the electron density, $V_{elec}^{AB}$, and a non-classical or exchange-correlation one, $V_{XC}^{AB}$, as shown in Equation (4).

(4) $$V_{ee}^{AB}=V_{elec}^{AB}+V_{XC}^{AB}$$

Let us now isolate the $V_{XC}^{AB}$ term and include $V_{en}^{AB}$, $V_{ne}^{AB}$, $V_{nn}^{AB}$, and the classical contribution of $V_{ee}^{AB}$ ($V_{elec}^{AB}$) in the classical Coulombic energy, which is thus defines as indicated in Equation (5):

(5) $$V_{Coul}^{AB}=V_{en}^{AB}+V_{ne}^{AB}+V_{nn}^{AB}+V_{elec}^{AB}$$

The classical Coulomb contribution for the intra-atomic terms is analogous, see Equation (6).

(6) $$V_{Coul}^{AA}=V_{en}^{AA}+V_{ne}^{AA}+V_{elec}^{AA}$$

As a result, intra- and inter-atomic energy contributions can be expressed according to Equations (7) and (8).

(7) $$E_{intra}^{AA}=T_{A}+V_{Coul}^{AA}+V_{XC}^{AA}$$

(8) $$E_{inter}^{AB}=V_{Coul}^{AB}+V_{XC}^{AB}$$

Since IQA is based on a given exhaustive partition of space, it can also be applied to ELF partitions. We have already shown how this can be done [13]. Notice that this allows us to include some ELF basins related to electron pairs as lone pairs (monosyaptic basins) and bonds (disynaptic and higher order) in addition to atomic-like contributions (the core basins). In this regard, it is necessary to highlight that the former lack nuclei in their interior, so that there are no intra-domain electron-nuclear potential terms for them.

Since we are interested in interactions between C–C bonds, let us take a general V(C_i_,C_j_) disynaptic basin, and label it as “Bond”. Given that this basin does not involve any nucleus: $V_{en}^{AA}=V_{en}^{Bond}=0$, and $E_{intra}^{Bond}$ will read as shown in Equation (9).

(9) $$E_{intra}^{Bond}=T^{Bond}+V_{Coul}^{Bond}+V_{XC}^{Bond}$$

For the “Bond” basin interaction with the core basin of atom A, for instance, it turns out that $V_{en}^{A\text{-}Bond}$ and $V_{nn}^{A\text{-}Bond}$ vanish, and the *A*-*Bond* interaction can be calculated according to Equation (10).

(10) $$E_{inter}^{A\text{-}Bond}=V_{ne}^{A\text{-}Bond}+V_{Coul}^{A\text{-}Bond}+V_{XC}^{A\text{-}Bond}$$

Finally, the interaction between two basins representing two different bonds, say “*Bond1*” and “*Bond2*” is given by Equation (11). Notice that, in this case, since any of the interaction entities bear any nucleus: $V_{en}^{Bond1\text{-}Bond2}=V_{nn}^{Bond1\text{-}Bond2}=V_{en}^{Bond1\text{-}Bond2}=V_{nn}^{A\text{-}Bond}=0$, that is to say, the only non-vanishing term is $V_{ee}^{Bond1\text{-}Bond2}$.

(11) $$E_{inter}^{Bond1\text{-}Bond2}=V_{Coul}^{Bond1\text{-}Bond2}+V_{XC}^{Bond1\text{-}Bond2}$$

From the previous discussion, it is clear that the use of IQA-ELF leads to an energy decomposition in which the basic interacting units are electron pairs, thus leading to interactions between cores and bonds, or between the possible combinations of bonds and lone pairs. IQA-ELF calculations have been performed with a modified version of the PROMOLDEN code that integrates over ELF instead of over QTAIM basins [13]. ELF representations were performed by means of UCSF Chimera package [67,68].

A set of 28 mono-substituted benzene derivatives (Ph–X) have been optimized at the above-mentioned level of theory. These include inductively (−I) and resonance mediated (−M) electron withdrawing groups (EWG), as well as inductively (+I) and resonance mediated (+M) electron donating groups (EDG). The list includes, in addition to benzene, the Me, Et and Pr alkyl groups, the F, Cl, and Br halides, and the CHF_2_, CH_2_Cl, and CF_3_ species, the NO_2_, O^−,^ NH_2_, NH_3_^+^, SH, SiH_3_, SiH_2_Me, CN, CCH, CC–F, CC–Cl, CC–Me, and Ph groups, and also a number of non-standard substituents like Li, BH_2_, BMe_2_, BF_2_, and AlH_2_. Common wisdom establishes that mesomeric (M) effects are generally stronger than inductive ones, and that EWGs deactivate the phenyl ring toward electrophilic attack by decreasing its electron density while EDGs activate it. Usually, activation and deactivation is stronger at the ortho/para positions (especially when it is due to mesomeric effects), meaning that, in general, deactivating groups direct electrophiles to the meta position [31]. Note that we did not include OH and OMe substituents in our survey due to integration issues generated by the irregular shape of the V(C,O) basin, which led to rather large errors with the PROMOLDEN code for these two substituents.

## 4. Conclusions

In this contribution, we have shown that inductive and mesomeric effects can be grasped from the behavior of the population of the aromatic valence bond basins. Dominating inductive effects decay exponentially with the distance to the perturbing center, as in insulators, while resonance or mesomeric effects show an oscillatory pattern that recalls known behavior in metallic systems.

Delocalization indices between adjacent C–C bonds provided by the IQA-ELF framework have been found as particularly suited expectation values to that end. In agreement with chemical intuition, when a substituent increases the electron flow ability between the two aromatic bonds that share a given carbon atom of the ring, the position tends to be activated toward electrophilic attack and vice versa. This good agreement strongly supports the good performance of IQA-ELF methodology in capturing the chemical behavior and making accurate predictions. Moreover, we have convincingly shown that the basin kinetic energy evolves in consonance with basin populations, and that exchange-correlation energies provide a similar energetic analog to the population fluctuations.

The relevance of this work is double, as, on the one hand, we have provided a new energetic descriptor for the prediction of EAS reactions based on the ELF topology. On the other hand, we have proven that the IQA-ELF scheme works well in predicting chemical reactivity. This will be very useful for paving the way to the construction of well-behaved electron-pair energetic descriptors to be used in future force field developments, as they accurately capture the chemical behavior of the systems under study.

## Figures and Tables

**Table 1:** Classification of X substituents according to their behavior against aromatic electrophilic substitution. Activating/deactivating groups are marked by +/− signs, respectively, and the *e*, *s*, *m*, and *w* labels refer to extreme, strong, medium or weak effects. Similarly, I and M refer to inductive and mesomeric effects, and +/− signals whether the group donates or extracts electronic charge from the ring.

X.	Activation	Electronic Effect	Directing Effect
O^−^	*+e*	+I/+M	*o/p*
NH_2_	*+s*	−I/+M	*o/p*
SH	*+m*	−I/+M	*o/p*
CH_3_,R	*+w*	+I	*o/p*
F ^[a]^	*+w*	−I/+M	*p*
F ^[a]^	*−w*	−I/+M	*o*
Cl,Br,I	*−w*	−I/+M	*o/p*
NO_2_,CN	*−s*	−I/−M	*m*
CF_3_,NH_3_^+^	*−s*	−I	*m*

^[a]^ F is understood as a weak activating substituent at the *para* position and a weak deactivating one at the *ortho* position.

## Data Availability

Not applicable.

## Supplementary Materials

The following are available online, Table S1. ELF basin populations, Table S2. Delocalization indices relative to benzene, Table S3. Kinetic energy, Table S4. Exchange-correlation energies relative to benzene, Table S5. Cartesian coordinates, Figure S1. ELF basin populations relative to benzene, Figure S2. Kinetic energy with relative to benzene.

## Sample Availability

Samples of compounds are not available from the authors.
