# Supplementary material for: Energetics of Electron Pairs in Electrophilic Aromatic Substitutions
Source: Molecules. 2021 Jan 19;26(2):513. doi: 10.3390/molecules26020513 (PMC7835785; doi:10.3390/molecules26020513)
Supplement: Supplementary file 1 [file molecules-26-00513-s001.pdf]

Supporting Information for

Energetics of electron pairs in Electrophilic Aromatic Substitutions

Julen Munárriz[1,*], Miguel Gallegos[1], Julia Contreras-García [2] and Ángel Martín Pendás[1,*]

[1] Departamento de Química Física y Analítica. Universidad de Oviedo, 33006 Oviedo Spain; munarrizjulen@uniovi.es (J.M.), gallegosmiguel@uniovi.es (M.G), ampendas@uniovi.es (A.M.P).
[2] Sorbonne Université, CNRS, Laboratoire de Chimie Théorique, LCT, F. 75005 Paris, France; contreral@lct.jussieu.fr (J.C.-G.)
* Correspondence: munarrizjulen@uniovi.es, ampendas@uniovi.es

Table of Contents

ELF basin populations (Table S1) S2

ELF basin populations relative to benzene (Figure S1) S3

Delocalization indices relative to benzene (Table S2) S4

Kinetic energy (Table S3) S5

Kinetic energy with relative to benzene (Figure S2) S6

Exchange-correlation energies relative to benzene (Table S4) S7

Cartesian coordinates (Table S5) S8

ELF basin populations

Table S1. *bij* basin populations (*nij*) for the complete set of Ph-X systems. All data in au.

X	*n12*	*n23*	*n34*	Average
H	2.79	2.79	2.79	2.79
O^-	2.49	3.29	2.64	2.80
NH_2	2.73	2.96	2.81	2.83
SH	2.75	2.89	2.78	2.82
CH_3	2.83	2.80	2.79	2.81
F	2.86	2.86	2.81	2.84
Br	2.85	2.84	2.79	2.83
NO_2	2.86	2.81	2.75	2.81
CN	2.74	2.84	2.76	2.78
CF_3	2.82	2.80	2.77	2.80
NH_3^+	2.92	2.79	2.75	2.80
BH_2	2.55	2.84	2.73	2.70
Li	2.59	2.81	2.79	2.73
Et	2.82	2.81	2.79	2.81
Pr	2.82	2.81	2.79	2.81
CHF_2	2.82	2.80	2.78	2.80
CH_2Cl	2.80	2.81	2.78	2.80
Cl	2.86	2.84	2.79	2.83
SiH_3	2.66	2.81	2.77	2.75
SiH_2Me	2.66	2.82	2.77	2.75

CCH	2.72	2.87	2.77	2.79
AlH_2	2.55	2.82	2.75	2.71
Ph	2.78	2.83	2.78	2.79
BF2	2.62	2.82	2.75	2.73
BMe_2	2.59	2.82	2.74	2.72
CC-F	2.73	2.86	2.77	2.79
CC-Cl	2.72	2.87	2.77	2.78
CC-Me	2.72	2.87	2.77	2.79

ELF basin populations with respect to benzene

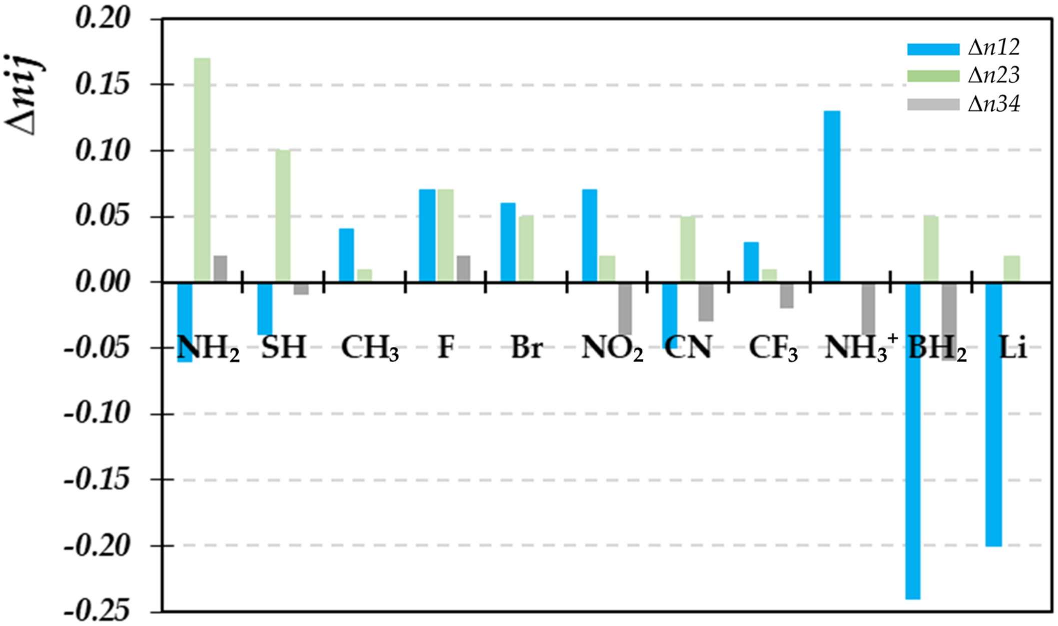

Figure S1. *bij* basin populations with respect to benzene (Δ*nij*) for the selected set of Ph-X systems commented in the main text. All data in au. We have excluded X = O^- for visual purposes (Δ*n12* = -0.30, Δ*n23* = 0.50, Δ*n34* = -0.15).

Delocalization Indices with respect to benzene

Table S2. Vicinal delocalization indices, ΔDI(a), in the aromatic ring basins for the full set of molecules. All data in au $\times 10^{-3}$, referred to the DI of adjacent carbon bonds in benzene, equal to 0.555 au.

X	ΔDI(*i*)	ΔDI(*o*)	ΔDI(*m*)	ΔDI(*p*)
O^-	-147.1	57.7	-4.6	-111.9
NH_2	-34.3	38.2	-3.1	22.0
SH	-8.2	9.9	-3.0	7.2
CH_3	1.6	7.8	-2.5	3.5
F	21.6	31.9	-4.1	13.7
Br	22.2	9.6	-1.5	0.6
NO_2	37.9	-13.6	2.9	-20.3
CN	-11.3	-16.8	3.7	-16.1
CF_3	17.8	-9.3	-0.5	-8.9
NH_3^+	76.2	7.4	-15.0	-18.2

BH_2	-148.1	-48.4	12.0	-32.4
Li	-140.7	-16.6	3.5	1.4
Et	-4.5	6.6	-2.2	3.8
Pr	-5.0	5.9	-2.6	3.6
CHF_2	13.9	-5.3	-1.9	-5.3
CH_2Cl	-2.0	-5.3	-0.3	-5.6
Cl	30.3	11.7	-1.3	1.5
SiH_3	-80.6	-21.9	2.3	-9.9
SiH_2Me	-87.1	-21.4	2.7	-7.8
CCH	-23.5	-8.9	4.5	-9.6
AlH_2	-145.3	-38.8	6.3	-18.9
Ph	-16.4	-2.5	-1.0	-3.8
BF_2	-103.6	-34.8	6.8	-22.2
BMe_2	-129.3	-37.1	8.5	-22.4
CC-F	-19.9	-6.5	3.6	-8.2
CC-Cl	-24.9	-8.9	4.2	-10.1
CC-Me	-25.4	-6.2	4.2	-7.2

Kinetic energy

Table S3. *bij* basin kinetic energies (*tij*) for the full set of systems. Notation as in Table 2 in the main text. All data in au.

X	*t12*	*t23*	*t34*	Average
H	3.254	3.254	3.254	3.254
O^-	2.985	3.678	3.112	3.259
NH_2	3.259	3.407	3.275	3.314
SH	3.274	3.354	3.255	3.294
CH_3	3.305	3.27	3.255	3.277
F	3.470	3.322	3.27	3.354
Br	3.368	3.309	3.251	3.309
NO_2	3.412	3.292	3.219	3.307
CN	3.225	3.328	3.221	3.258
CF_3	3.330	3.260	3.245	3.279
NH_3^+	3.464	3.265	3.214	3.314
BH_2	3.011	3.304	3.190	3.168
Li	3.007	3.280	3.243	3.177
Et	3.293	3.283	3.255	3.277
Pr	3.294	3.279	3.257	3.277
CHF_2	3.312	3.257	3.248	3.272
CH_2Cl	3.294	3.268	3.251	3.271
Cl	3.387	3.312	3.251	3.317
SiH_3	3.111	3.276	3.235	3.208
SiH_2Me	3.097	3.274	3.248	3.206
CCH	3.212	3.344	3.228	3.261
AlH_2	2.993	3.297	3.213	3.168

Ph	3.263	3.290	3.246	3.266
BF2	3.081	3.294	3.208	3.194
BMe_2	3.050	3.294	3.218	3.187
CC-F	3.224	3.344	3.229	3.266
CC-Cl	3.208	3.349	3.226	3.261
CC-Me	3.212	3.349	3.230	3.264

Kinetic energy relative to benzene

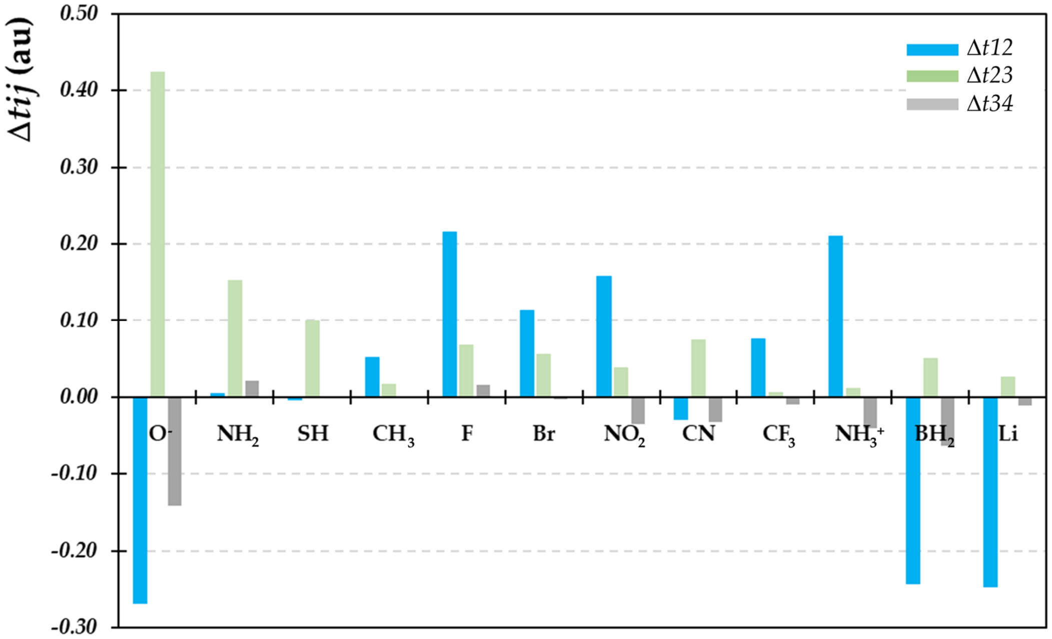

Figure S2. *bij* basin kinetic energies relative to benzene (Δ*tij*) for the selected set of Ph-X systems commented in the main text. All data in au.

Exchange-correlation energies relative to benzene

Table S4. Vicinal exchange-correlation energies relative to benzene (ΔV_{xc}) for the full set of molecules. See the main text for a description. All data in kcal/mol, referred to the vicinal V_{xc} value in benzene, equal to -100.4 kcal/mol.

X	$\Delta V_{xc}(i)$	$\Delta V_{xc}(o)$	$\Delta V_{xc}(m)$	$\Delta V_{xc}(p)$
O^-	21.3	-11.9	-0.534	18.2
NH_2	2.49	-6.87	0.687	-3.78
SH	-1.25	-2.17	0.799	-1.26
CH_3	-0.963	-1.05	0.159	-0.729
F	-1.37	-3.05	4.25	-0.346
Br	-6.43	-1.83	0.492	-0.066
NO_2	-9.41	2.32	-0.110	2.99
CN	-0.878	2.52	-0.201	2.37
CF_3	-4.81	1.46	0.343	1.28
NH_3^+	-16.6	-1.29	3.05	3.56
BH_2	22.6	6.80	-1.59	4.32

Li	22.7	1.46	-0.985	-0.859
Et	-0.025	-0.986	0.177	-0.884
Pr	0.031	-0.876	0.289	-0.816
CHF_2	-3.70	0.898	0.429	0.658
CH_2Cl	-1.39	0.945	0.176	0.632
Cl	-7.78	-2.11	0.447	-0.196
SiH_3	12.3	2.79	-0.230	1.23
SiH_2Me	13.5	2.69	-0.340	0.763
CCH	1.29	1.20	-0.434	1.19
AlH_2	22.6	4.84	-0.887	2.44
Ph	1.28	0.499	0.167	0.379
BF_2	15.3	4.70	-0.850	3.05
BMe_2	20.0	5.22	-1.27	2.74
CC-F	0.779	0.85	-0.332	0.983
CC-Cl	1.47	1.15	-0.350	1.28
CC-Me	1.65	0.835	-0.400	0.769

Cartesian Coordinates

Table S5. xyz coordinates (in bohr) for the optimized geometries of all the molecules considered in this work (Ph-X).

X = H

6 0.000179 -1.396578 -0.000093
6 1.209602 -0.698085 -0.000040
6 1.209464 0.698416 0.000043
6 -0.000179 1.396706 0.000085
6 -1.209643 0.698106 0.000043
6 -1.209423 -0.698395 -0.000040
1 2.150994 -1.241780 -0.000070
1 2.150932 1.241840 0.000080
1 -0.000318 2.483668 0.000154
1 -2.151250 1.241290 0.000079
1 -2.150676 -1.242331 -0.000070
1 0.000318 -2.483711 -0.000157

X = O$^-$

6 -1.084352 0.000000 -0.000037
6 -0.286381 1.213131 0.000001
6 1.102020 1.201106 -0.000005
6 1.830814 0.000000 -0.000004
6 1.102020 -1.201106 0.000016
6 -0.286381 -1.213131 -0.000002
1 -0.832838 2.156953 0.000025
1 1.642376 2.152193 0.000000
1 2.919897 0.000000 -0.000002
1 1.642376 -2.152193 0.000026
1 -0.832838 -2.156953 0.000006
8 -2.350677 0.000000 0.000017

X = NH2

6 0.938719 -0.000041 -0.010145
6 0.221274 1.208272 -0.005203
6 -1.171806 1.202652 0.003385
6 -1.881669 0.000007 0.008366
6 -1.171860 -1.202617 0.003359
6 0.221261 -1.208281 -0.005196
1 0.763329 2.151748 -0.013398
1 -1.705683 2.149782 0.008807
1 -2.967630 0.000051 0.016695
1 -1.705716 -2.149760 0.008745
1 0.763246 -2.151800 -0.013300
7 2.337402 -0.000002 -0.078895
1 2.777543 0.834924 0.288615
1 2.777583 -0.834877 0.288702

X = SH

6 0.506804 -0.000198 -0.000039
6 -0.201602 -1.209423 -0.000071
6 -1.595546 -1.201760 -0.000025
6 -2.298111 0.004804 0.000035
6 -1.591283 1.207831 0.000025
6 -0.196050 1.211041 0.000002
1 0.335869 -2.153977 -0.000204
1 -2.133216 -2.146231 -0.000031
1 -3.384249 0.007202 0.000075
1 -2.124816 2.154754 0.000040

1 0.340605 2.156090 0.000067
16 2.293903 -0.083608 0.000105
1 2.518082 1.246117 -0.001195

X = CH_3
6 -0.914018 0.000032 -0.011139
6 -0.194225 -1.202283 -0.008864
6 1.201268 -1.205486 0.002163
6 1.905062 -0.000022 0.008406
6 1.201323 1.205456 0.002163
6 -0.194186 1.202305 -0.008866
1 -0.734317 -2.146857 -0.017698
1 1.738955 -2.150290 0.001414
1 2.991880 -0.000046 0.013688
1 1.739042 2.150242 0.001414
1 -0.734244 2.146894 -0.017695
6 -2.425683 0.000014 0.009226
1 -2.834054 -0.885153 -0.490551
1 -2.810495 -0.001926 1.038152
1 -2.834020 0.887038 -0.487252

X = F
6 0.931557 0.000004 -0.000024
6 0.260449 1.217592 0.000058
6 -1.135579 1.208706 -0.000057
6 -1.835846 -0.000001 -0.000003
6 -1.135572 -1.208711 0.000073
6 0.260449 -1.217590 -0.000064
9 2.282496 -0.000001 0.000012
1 0.828684 2.142022 0.000081
1 -1.675344 2.151646 -0.000065
1 -2.921909 -0.000008 -0.000012
1 -1.675343 -2.151648 0.000092
1 0.828699 -2.142011 -0.000097

X = Br
6 -0.104266 0.000001 0.000015
6 -0.785169 1.215825 0.000010
6 -2.181348 1.207844 -0.000008
6 -2.881397 -0.000001 -0.000005
6 -2.181346 -1.207846 0.000004
6 -0.785169 -1.215823 -0.000004
35 1.810984 0.000000 -0.000002
1 -0.233202 2.149453 0.000024
1 -2.719130 2.152011 -0.000021
1 -3.967620 -0.000002 -0.000009
1 -2.719128 -2.152013 0.000016
1 -0.233194 -2.149448 -0.000003

X = NO_2
6 -0.244824 0.000021 -0.000016
6 0.427781 -1.220751 -0.000127
6 1.820975 -1.212340 -0.000094
6 2.516514 0.000006 0.000029
6 1.821086 1.212335 0.000129
6 0.427817 1.220783 0.000120
1 -0.140231 -2.142803 -0.000246
1 2.363133 -2.153181 -0.000162
1 3.602876 -0.000087 0.000047
1 2.363225 2.153189 0.000213
1 -0.140051 2.142894 0.000218
7 -1.717857 0.000057 -0.000037
8 -2.290143 1.089788 0.000377
8 -2.289863 -1.089881 -0.000383

X = CN
6 -0.610440 0.000167 -0.000007
6 0.091612 -1.217354 0.000044
6 1.483864 -1.210952 0.000058
6 2.180727 0.000007 0.000021
6 1.484080 1.210834 -0.000031
6 0.091589 1.217347 -0.000044
1 -0.459219 -2.152459 0.000071
1 2.025879 -2.152105 0.000098
1 3.267140 -0.000079 0.000032
1 2.026021 2.152028 -0.000060
1 -0.458775 2.152736 -0.000083
6 -2.045059 -0.000270 -0.000019
7 -3.208470 0.000172 -0.000028

X = CF_3
6 0.049324 -0.000005 -0.034500
6 0.745085 -1.212050 -0.022342
6 2.139180 -1.209396 0.002219
6 2.836988 -0.000010 0.015397
6 2.139171 1.209395 0.002207
6 0.745097 1.212052 -0.022363
1 0.197322 -2.148566 -0.039928
1 2.679848 -2.151562 0.007656
1 3.923421 0.000011 0.033065
1 2.679873 2.151542 0.007634
1 0.197315 2.148561 -0.039964
6 -1.455010 0.000019 -0.002045
9 -1.973233 1.090073 -0.611276
9 -1.928708 0.000034 1.266983
9 -1.973258 -1.090109 -0.611253

X = NH_3^+

6 -0.826535 0.000793 -0.007992
6 -0.167492 1.223782 -0.004316
6 1.228945 1.212273 0.001264
6 1.920560 -0.000549 0.004533
6 1.226424 -1.213321 0.001314
6 -0.169016 -1.224277 -0.004313
1 -0.710920 2.165642 -0.006706
1 1.769738 2.152844 0.000465
1 3.005799 -0.001549 0.006952
1 1.766634 -2.154269 0.000545
1 -0.713835 -2.165082 -0.007047
7 -2.325644 0.000215 0.006115
1 -2.699039 0.915341 -0.276219
1 -2.710799 -0.697614 -0.643944
1 -2.705390 -0.209014 0.940207

X = BH_2
6 1.018800 0.000241 0.000004
6 0.285346 1.208517 0.000003
6 -1.106975 1.213235 -0.000080
6 -1.802262 -0.000133 -0.000001
6 -1.106823 -1.213366 0.000065
6 0.285477 -1.208268 0.000002
1 0.827317 2.151229 0.000111
1 -1.653251 2.152752 -0.000160
1 -2.889575 -0.000275 0.000016
1 -1.653000 -2.152966 0.000166
1 0.827628 -2.150874 -0.000111
5 2.554910 -0.000065 0.000052
1 3.161854 -1.032797 -0.000735
1 3.163099 1.031899 0.000497

X = Li
6 1.280143 0.000003 -0.000006
6 0.515424 1.191150 -0.000002
6 -0.883540 1.204008 0.000002
6 -1.590280 -0.000003 0.000002
6 -0.883535 -1.204011 -0.000002
6 0.515428 -1.191148 -0.000006
1 1.021118 2.160256 -0.000003
1 -1.423706 2.149942 0.000004
1 -2.678483 -0.000005 0.000006
1 -1.423697 -2.149947 -0.000002
1 1.021127 -2.160251 -0.000010
3 3.253936 0.000001 0.000025

X = Et
6 0.434894 -0.000131 -0.328628
6 -0.270166 1.202595 -0.185279
6 -1.637046 1.205979 0.095843
6 -2.325920 0.000121 0.238248
6 -1.637259 -1.205837 0.095987
6 -0.270341 -1.202701 -0.185144
1 0.258438 2.146989 -0.299829
1 -2.165002 2.150709 0.198839
1 -3.391174 0.000254 0.453552
1 -2.165317 -2.150497 0.199091
1 0.258070 -2.147214 -0.299562
6 1.926557 -0.000210 -0.592048
1 2.193400 -0.879513 -1.192099
1 2.193424 0.878755 -1.192594
6 2.762265 0.000149 0.700795
1 3.834900 0.000153 0.474501
1 2.542632 0.884518 1.309510
1 2.542732 -0.883940 1.309952

X= Pr
6 0.121695 -0.000081 0.371724
6 0.820827 1.202590 0.200488
6 2.175607 1.205935 -0.134166
6 2.858376 0.000088 -0.303364
6 2.175732 -1.205839 -0.134273
6 0.820947 -1.202662 0.200383
1 0.297156 2.146948 0.335770
1 2.699067 2.150665 -0.258026
1 3.914355 0.000155 -0.560348
1 2.699284 -2.150507 -0.258215
1 0.297374 -2.147085 0.335578
6 -1.357839 -0.000162 0.692952
1 -1.603996 -0.879859 1.302986
1 -1.604013 0.879271 1.303361
6 -2.250876 0.000099 -0.564085
1 -2.004747 0.877636 -1.176740
1 -2.004737 -0.877179 -1.177107
6 -3.745834 0.000023 -0.232023
1 -4.355943 0.000214 -1.142589
1 -4.022799 -0.884942 0.354571
1 -4.022805 0.884736 0.354947

X = CHF_2
6 0.178152 -0.000014 -0.151539
6 -0.519353 1.210176 -0.088343
6 -1.908418 1.208446 0.038186
6 -2.605053 0.000008 0.100499
6 -1.908442 -1.208428 0.038244
6 -0.519365 -1.210179 -0.088290
1 0.026643 2.147304 -0.127710
1 -2.445948 2.151172 0.092750

1 -3.687210 0.000020 0.199484
1 -2.445971 -2.151153 0.092867
1 0.026607 -2.147319 -0.127648
6 1.673058 0.000040 -0.343805
1 1.976012 0.000005 -1.398116
9 2.233668 -1.108482 0.235415
9 2.233708 1.108446 0.235547

X = CH_2Cl
6 -0.021229 -0.000091 0.445051
6 0.659838 -1.207151 0.241067
6 1.996059 -1.208256 -0.157275
6 2.666839 0.000076 -0.357239
6 1.995937 1.208355 -0.157172
6 0.659745 1.207079 0.241172
1 0.138031 -2.149036 0.393409
1 2.513972 -2.151266 -0.309575
1 3.708741 0.000150 -0.665835
1 2.513793 2.151406 -0.309410
1 0.137821 2.148880 0.393629
6 -1.455817 -0.000128 0.879746
1 -1.709432 0.889557 1.457493
1 -1.709482 -0.889924 1.457283
17 -2.623628 0.000054 -0.542888

X = Cl
6 0.503962 0.000003 0.000003
6 -0.178617 1.215754 0.000003
6 -1.574703 1.207600 -0.000002
6 -2.274992 -0.000001 -0.000002
6 -1.574693 -1.207605 0.000002
6 -0.178616 -1.215752 0.000001
17 2.265049 0.000001 -0.000002
1 0.373660 2.149455 0.000012
1 -2.113016 2.151285 -0.000006
1 -3.361196 -0.000006 -0.000006
1 -2.113013 -2.151287 -0.000003
1 0.373675 -2.149447 0.000003

X = SiH_3
6 -0.468423 -0.000020 -0.012632
6 0.256627 -1.205137 -0.010088
6 1.652109 -1.207656 0.003357
6 2.352273 0.000019 0.010977
6 1.652084 1.207666 0.003354
6 0.256589 1.205109 -0.010090
1 -0.272024 -2.156069 -0.023802
1 2.192337 -2.150924 0.003692
1 3.439182 0.000030 0.018554
1 2.192283 2.150950 0.003687
1 -0.272104 2.156013 -0.023804
14 -2.347617 -0.000001 0.006121
1 -2.863858 -1.212692 -0.685494
1 -2.893223 -0.002654 1.393053
1 -2.863501 1.215468 -0.680852

X = SiH_2Me
6 0.045269 -0.000661 -0.263806
6 -0.670006 1.203907 -0.134720
6 -2.041433 1.208062 0.124036
6 -2.730057 0.000857 0.256173
6 -2.042356 -1.207096 0.126189
6 -0.670915 -1.204446 -0.132576
1 -0.152181 2.154953 -0.244020
1 -2.573202 2.151624 0.217681
1 -3.798761 0.001443 0.454862
1 -2.574837 -2.150087 0.221518
1 -0.153789 -2.156059 -0.240176
14 1.903717 -0.001665 -0.564701
1 2.257046 1.205974 -1.365320
1 2.256523 -1.213742 -1.358805
6 2.902001 0.002538 1.041017
1 2.677115 -0.881094 1.648845
1 3.977678 0.001835 0.829286
1 2.677353 0.889493 1.644077

X = CCH
6 -0.594106 0.000016 0.000009
6 0.119909 1.213144 0.000040
6 1.512552 1.208607 -0.000005
6 2.212960 -0.000017 -0.000055
6 1.512525 -1.208625 -0.000006
6 0.119881 -1.213128 0.000040
1 -0.428533 2.150028 0.000108
1 2.052996 2.151314 0.000016
1 3.299550 -0.000029 -0.000107
1 2.052946 -2.151345 0.000016
1 -0.428583 -2.150001 0.000108
6 -2.024169 0.000015 0.000011
6 -3.234213 -0.000001 -0.000025
1 -4.300410 -0.000031 -0.000192

X = AlH_2
6 0.540623 -0.000122 -0.000053
6 -0.195082 1.204975 -0.000036
6 -1.590394 1.209572 0.000014
6 -2.289225 0.000132 0.000032
6 -1.590598 -1.209476 -0.000008

6 -0.195325 -1.205133 -0.000009
1 0.331297 2.158171 -0.000038
1 -2.133359 2.151565 0.000044
1 -3.376469 0.000209 0.000058
1 -2.133791 -2.151337 -0.000016
1 0.330896 -2.158417 -0.000039
13 2.489334 -0.000053 0.000024
1 3.270680 -1.386559 0.000048
1 3.269401 1.387376 -0.000014

X = Ph
6 -0.742882 0.000042 0.000040
6 -1.465802 1.138771 0.394210
6 -2.859944 1.139458 0.393710
6 -3.563829 -0.000015 -0.000006
6 -2.859869 -1.139504 -0.393755
6 -1.465779 -1.138761 -0.394205
1 -0.928550 2.022014 0.728540
1 -3.397114 2.029730 0.710496
1 -4.650493 -0.000077 -0.000035
1 -3.397080 -2.029745 -0.710553
1 -0.928399 -2.021919 -0.728540
6 0.742880 0.000002 0.000028
6 1.465770 -1.138747 0.394224
6 1.465813 1.138782 -0.394195
6 2.859913 -1.139477 0.393727
1 0.928480 -2.021952 0.728577
6 2.859901 1.139479 -0.393739
1 0.928471 2.021972 -0.728504
6 3.563831 -0.000027 -0.000022
1 3.397067 -2.029754 0.710512
1 3.397116 2.029719 -0.710538
1 4.650493 -0.000013 -0.000054

X = BF$_2$
6 0.192218 -0.000119 0.000002
6 -0.527005 1.209253 0.000015
6 -1.920434 1.211662 0.000013
6 -2.616890 0.000064 -0.000002
6 -1.920570 -1.211604 -0.000013
6 -0.527136 -1.209369 -0.000010
1 0.015326 2.151099 0.000036
1 -2.464715 2.152212 0.000016
1 -3.703964 0.000104 -0.000009
1 -2.464965 -2.152090 -0.000014
1 0.015086 -2.151283 -0.000026
5 1.737450 -0.000002 -0.000005
9 2.435391 -1.132747 0.000027
9 2.435153 1.132819 -0.000028

X = BMe$_2$
6 0.137061 0.000399 0.000269
6 -0.603451 -1.200651 -0.035847
6 -1.997069 -1.207553 -0.043149
6 -2.697824 -0.000042 -0.000190
6 -1.997453 1.207655 0.043003
6 -0.603803 1.201194 0.036179
1 -0.076407 -2.150321 -0.064461
1 -2.537867 -2.149971 -0.077978
1 -3.785119 -0.000208 -0.000356
1 -2.538543 2.149913 0.077659
1 -0.077109 2.151036 0.064969
5 1.705606 0.000381 0.000360
6 2.534800 -1.347254 0.072516
1 2.798760 -1.636275 -0.958771
1 3.492127 -1.212600 0.591976
1 2.021075 -2.206135 0.517374
6 2.536408 1.346782 -0.072604
1 2.019600 2.211385 -0.502373
1 2.818145 1.625270 0.956844
1 3.485290 1.212823 -0.607744

X = CC-F
6 -0.007929 -0.000021 0.000077
6 0.706658 1.212362 -0.000115
6 2.099435 1.208074 0.000057
6 2.800605 0.000030 0.000066
6 2.099477 -1.208041 0.000057
6 0.706703 -1.212380 -0.000115
1 0.159033 2.149871 -0.000179
1 2.639180 2.151217 -0.000179
1 3.887143 0.000049 0.000054
1 2.639259 -2.151162 -0.000179
1 0.159108 -2.149907 -0.000179
6 -1.439915 -0.000054 0.000039
6 -2.644684 -0.000034 0.000071
9 -3.933981 0.000034 -0.000019

X = CC-Cl
6 0.530871 0.000228 -0.000165
6 1.245080 1.213669 -0.000020
6 2.637547 1.208461 0.000221
6 3.337750 -0.000313 0.000360
6 2.637091 -1.208819 0.000221
6 1.244622 -1.213487 -0.000021
1 0.697207 2.150872 -0.000181
1 3.178042 2.151086 0.000332
1 4.424269 -0.000519 0.000569
1 3.177229 -2.151649 0.000331

1 0.696391 -2.150481 -0.000182
6 -0.898272 0.000517 -0.000313
6 -2.108997 0.000316 -0.002935
17 -3.760429 -0.000161 0.000885

X = CC-Me

6 -0.035561 -0.000073 -0.000102
6 -0.753009 1.211926 0.000004
6 -2.145698 1.207944 0.000069
6 -2.847381 0.000064 0.000077
6 -2.145816 -1.207884 0.000066

1 4.465158 0.907559 -0.46943

6 -0.753127 -1.212001 0.000006
1 -0.205309 2.149431 0.000091
1 -2.685561 2.151201 0.000145
1 -3.934016 0.000118 0.000159
1 -2.685771 -2.151089 0.000141
1 -0.205515 -2.149545 0.000094
6 1.394306 -0.000117 -0.000250
6 2.606743 0.000252 -0.000520
6 4.066259 -0.000047 0.000208
1 4.465682 -0.046696 1.021429
1 4.465034 -0.861368 -0.549966
